# Correlation between hemodynamics assessed by FAI combined with CT-FFR and plaque characteristics in coronary artery stenosis

**DOI:** 10.1186/s12880-025-01590-8

**Published:** 2025-02-15

**Authors:** Bo Duan, Shuqing Deng, Runyang Xu, Yongsheng Wang, Kewu He

**Affiliations:** 1 Image Center, The Third Affiliated Hospital of Anhui Medical University (The First People’s Hospital of Hefei), Hefei, 230061 China; 2 Department of Psychology, Brandeis University, Waltham, MA 02453 USA; 3 Ultrasonography Lab, The First Affiliated Hospital of Anhui Medical University, Hefei, 230022 China; 4 Department of Cardiology, The Third Affiliated Hospital of Anhui Medical University (The First People’s Hospital of Hefei), Hefei, 230061 China

**Keywords:** Coronary artery disease, Coronary computed tomography angiography (CCTA), Pericoronary adipose tissue (PCAT), Fat attenuation index (FAI), CT derived fractional flow reserve (CT-FFR)

## Abstract

**Background:**

While both CT-FFR and FAI are found to be associated with the development of CAD, their relationship with hemodynamics and plaque characteristics remains unclear. The present study aims to investigate the relationship between hemodynamics assessed by FAI combined with CT-FFR and plaque characteristics in functionally significant coronary artery stenosis.

**Methods:**

This retrospective study included 130 patients with suspected coronary heart disease, who were admitted to the Department of Cardiology of our hospital and underwent coronary computed tomography angiography (CCTA) from January 2022 to December 2023. Clinical baseline data and relevant auxiliary examination results were collected, and CCTA, FAI, and CT-FFR data were analyzed to investigate the relationship between these imaging parameters and both the hemodynamics and plaque characteristics of coronary artery lesions.

**Results:**

From 130 patients, a total of 207 diseased vessels were analyzed and classified based on CAD-RADS grading: 128 vessels exhibited stenosis of less than 50%, and 79 exhibited stenosis exceeding 50%. Patients with more than one lesion of > 50% stenosis were classified into the myocardial ischemia group (44 cases), and the rest were categorized as the non-myocardial ischemia group (86 cases). Compared to the non-myocardial ischemia group, patients in the myocardial ischemia group were significantly older (*p* < 0.001). No significant difference was found between the two groups in sex, cardiovascular risk factors, or the indicator of stenotic vessel distribution. The minimum CT-FFR in vessels with < 50% stenosis was higher than in vessels with > 50% stenosis, ΔCT-FFR was lower in vessels with < 50% stenosis than in vessels with > 50% stenosis, and the median CT-FFR was significantly lower in vessels with > 50% stenosis than in vessels with < 50% stenosis (*p* < 0.001). Additionally, FAI-LAD, FAI-LCX, FAI-RCA, and FAI-Mean were found to be significantly higher in vessels with > 50% stenosis compared to vessels with < 50% stenosis (*p* < 0.05). A negative correlation was observed between the minimum CT-FFR among three main coronary arteries (LAD, LCX, RCA) and CAD-RADS classification, while both ΔCT-FFR and FAI were positively correlated with CAD-RADS classification (*p* < 0.05). Non-calcified plaques were more prevalent in the vessels with > 50% stenosis, primarily located in the LAD, while calcified plaques were predominantly observed in vessels with < 50% stenosis (*p* < 0.001). In addition, in vessels with > 50% stenosis, plaques were longer, the degree of luminal stenosis was greater, and both the total volume and burden of plaques were significantly greater than in vessels with < 50% stenosis (*p* < 0.001). Lastly, the FAI_lesion_ value in the vessels with > 50% stenosis was higher than in vessels with < 50% stenosis (*p* < 0.001).

**Conclusion:**

FAI is associated with coronary artery stenosis and myocardial ischemia, and may serve as a novel indicator for identifying myocardial ischemia. Both FAI and CT-FFR demonstrated strong predictive abilities in significant coronary stenosis.

## Background

As one of the most common cardiovascular diseases, Coronary artery disease (CAD) has a high incidence rate and is a major cause of mortality in patients [1]. CAD is caused by atherosclerosis, and the buildup and gradual progression of plaques inside arteries cause the narrowing or occlusion of coronary arteries, resulting in myocardial ischemia and myocardial infarction [2]. Past studies have suggested that both the formation of atherosclerosis and plaque rupture are closely related to vascular inflammation [3]. Therefore, early and accurate detection of coronary inflammation may improve the prognosis of CAD patients.

Among coronary artery imaging techniques, the invasive fractional flow reserve (FFR) is well-recognized in identifying myocardial ischemia caused by functional issues [4]. However, due to the complexity and high costs of the invasive procedure, the usage of FFR in clinical settings is less frequent. A non-invasive imaging technique, Coronary Computed Tomography Angiography (CCTA), is considered an effective method for identifying CAD. In addition to evaluating the degree of stenosis, CCTA is also crucial in predicting adverse clinical outcomes through assessing high-risk plaque features, including quantitative plaque features, levels of coronary inflammation, and indicators for blood vessel function [5]. Additionally, derived from CCTA, the CT-derived fractional flow reserve (CT-FFR) is calculated using computational fluid dynamics (CFD) and simulates coronary blood flow based on anatomic imaging data extracted from CCTA, providing CT-FFT at any location within the coronary tree without additional imaging or radiation exposure [6]. With further optimization of CT-FFR using CFD, researchers have confirmed its reliability as comparable to invasive FFR, and it demonstrates better accuracy in diagnosing hemodynamically significant lesions compared to CCTA alone [7].

Recently, the fat attenuation index (FAI) was introduced as a novel imaging biomarker to assess coronary artery inflammation in vivo, and due to the interaction between pericoronary adipose tissue (PCAT) and coronary plaques, an elevated FAI has been linked to increased risk of major adverse cardiovascular events [8, 9]. A study also suggested that, among patients with acute coronary syndrome, the FAI around culprit lesions is significantly higher compared to non-culprit lesions [10]. However, while both CT-FFR and FAI are found to be associated with the development of CAD [11–13], their relationship with hemodynamics and plaque characteristics remains unclear. The present study aims to investigate the relationship between FAI and hemodynamically significant coronary artery lesions assessed by CT-FFR and to evaluate the diagnostic value of combining FAI with CT-FFR in identifying coronary stenosis and myocardial ischemia.

## Method

### Data source and participants

Based on inclusion and exclusion criteria, this retrospective study included 130 patients with suspected CAD who underwent CCTA at the Department of Cardiology of our hospital between January 2022 and December 2023. Among 130 patients, 38 patients with at least one vessel stenosis of more than 50% and FFR < 0.8 underwent revascularization, and 92 patients were treated with medical statins and followed up for six months. The plaque and clinical manifestations of the patients were stable and partially improved. Subjects included both male and female patients, aged between 40 and 80 years old. Clinical baseline data and related auxiliary examination were collected, and CCTA, FAI, and CT-FFR data were analyzed. This study was approved by the hospital’s Ethics Committee (Ethics approval reference number: 2024–218-01).

### Inclusion/exclusion criteria

Inclusion criteria: 1) patients with angina or angina-like symptoms; 2) patients with intermediate risk of obstructive CAD, based on the Diamond-Forrester risk model; 3) at least one Coronary artery stenosis of 50% or more is found in CCTA; 4) Clear CCTA image with good contrast, without any artifact or misalignment.

Exclusion criteria: 1) past history of coronary revascularization; 2) past history of myocardial infarction; 3) patients with a low or high risk of obstructive CAD, based on the Diamond-Forrester risk model; 4) poor quality in CCTA image (moderate to severe artifacts); 5) patients with cardiomyopathy accompanied by other comorbidities; 6) failure in quantifications of pericoronary adipose tissue volume, FAI, CT-FFR, or plaque due to any reason.

### Imaging acquisition and analysis

#### CCTA image acquisition

All scans were performed on a GR Revolution CT scanner with 256 detector rows and 512 slices. Prior to the scans, each patient’s renal function and allergy history were reviewed to eliminate the risk of contrast allergy. Patients with baseline heart rates ≥ 65 bmp were given β-blockers (e.g., metoprolol 25–75 mg) one hour before the scan. Nitroglycerin (0.5 mg) was administered sublingually for all patients to dilate the coronary arteries. Correct placement of electrocardiogram electrodes was ensured, and parallel scans were performed from the tracheal carina to 2 cm below the diaphragmatic surface of the heart. Different acquisition protocols were applied based on the patient’s measured heart rate: for patients with a heart rate of less than 70 bpm, prospective ECG-gating technology was used, for patients with a heart rate of 70 bpm or higher, retrospective ECG-gating technology was applied.

#### CCTA image reconstruction and plaque quantitative and qualitative analysis

**Fig. 1:**
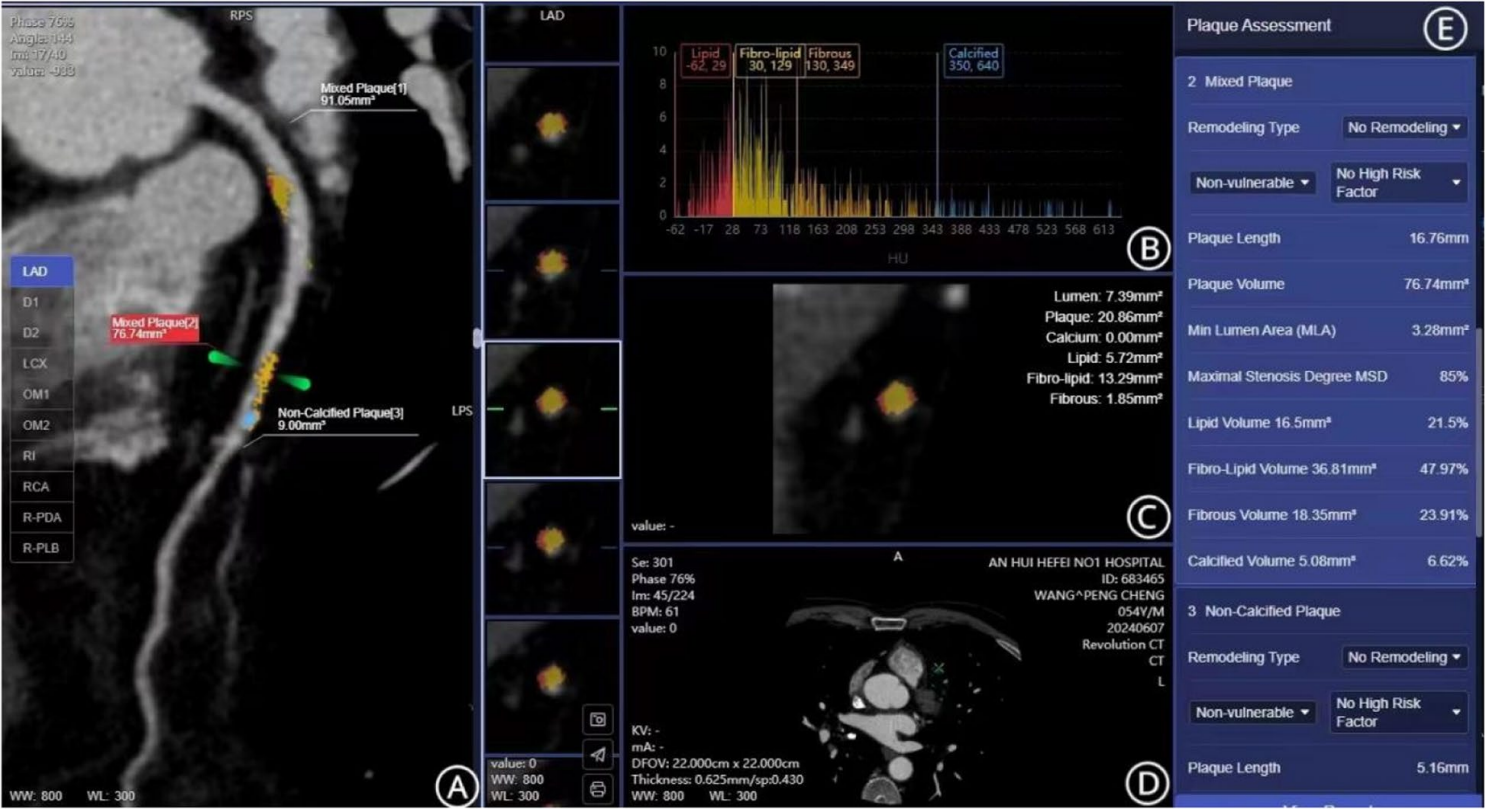
Quantitative Plaque Measurement (**A**: selecting the region of interest (ROI) for vascular plaque. **B**, **E**: analysis of plaque components and proportions. **C**, **D**: cross-sectional view of the vessel at a plaque’s position and components labeling)

All acquired CCTA images were transferred to the GE (AW4.7) post-processing workstation for analysis of coronary arteries with diameters greater than 2.0 mm. Based on CCTA results, both qualitative and quantitative plaque characteristics were analyzed. For the qualitative analysis, calcified plaques were defined as those with a CT value greater than 130 HU, distinguishable from the coronary artery lumen, and identifiable in more than two independent imaging slices. Non-calcified plaques were defined as those with a CT value of less than 130 HU but higher than connective tissue, identifiable in the coronary artery wall in more than two independent imaging slices. Mixed plaques contained both calcified and non-calcified components [14]. For the quantitative analysis, the coronary arteries of the heart were automatically extracted and marked using specialized software, including the LAD, LCX, RCA, D1, D2, R-PDA, and R-PLB. Locations of lesions in each vessel were manually annotated by technicians. A dual-source mode was used to trace the plaques, starting from the origin and extending to the endpoint. For vessels with multiple lesions, the most severe stenosis was selected as the representative lesion. Based on the determined lesion range, plaque length, plaque volume, size of minimum lumen area, degree of stenosis, and the volume and percentage of each plaque component (lipid component, fibrolipid component, fibrous component, and calcified component) were calculated automatically. The non-calcified volume was calculated as the sum of the lipid and fibrous volumes, and the plaque burden was calculated as plaque volume divided by vessel volume (Fig. 1). All parameters were examined independently by two experienced diagnostic physicians and the average values between the two observers were used for further analysis.

#### Volume and Fat Attenuation Index (FAI) of Pericoronary Adipose Tissue (PACT)

**Fig. 2:**
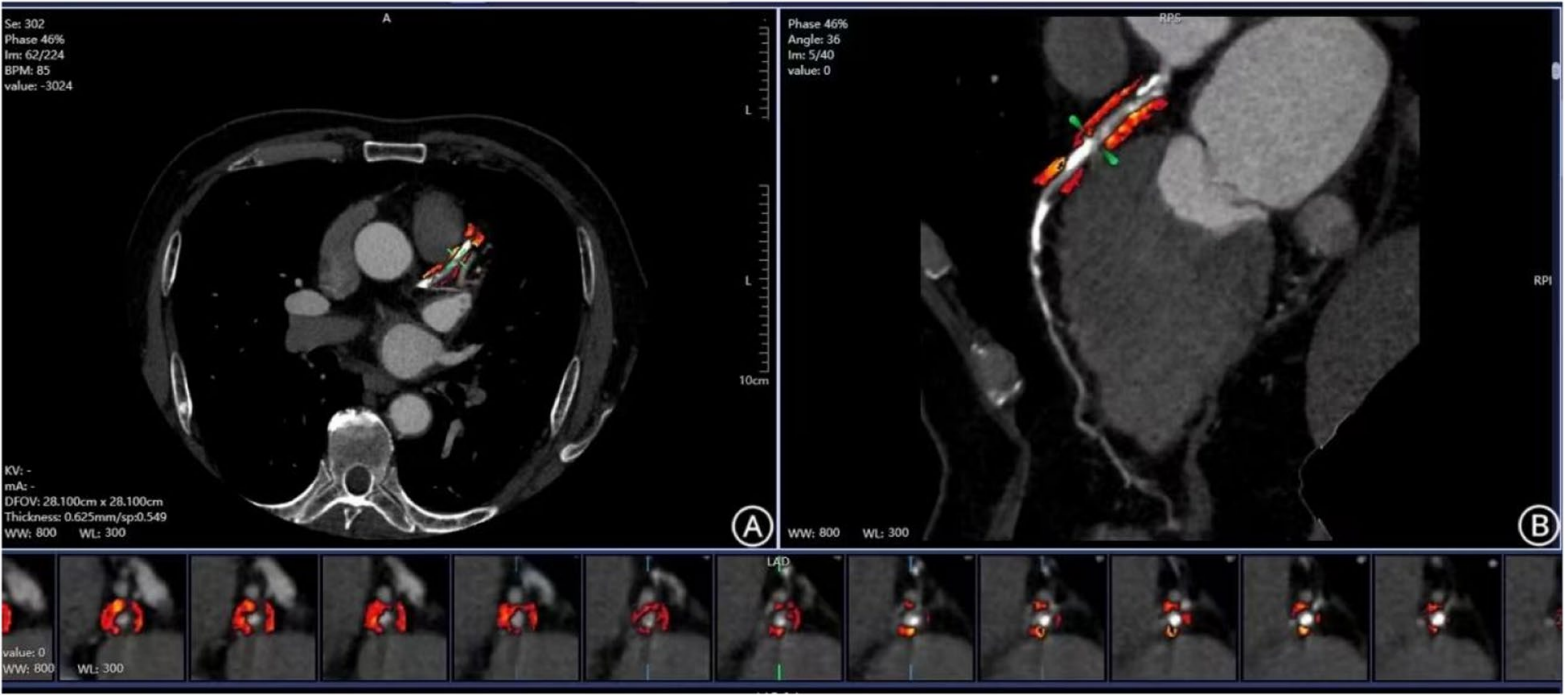
Measurement of the Volume and FAI of Pericoronary Adipose Tissue. Fig A shows cross-sectional images, suggesting calcified plaques in the proximal and middle segments of LAD; Fig B shows curved planar reconstruction images, suggesting severe vascular lumen stenosis, and orange is the area of pericoronary fat measurement

The SSF2-reconstructed CCTA images were uploaded to the GE (AW4.7) workstation, and images with the highest quality were sent to post-processing software (FFRDoc and EasyFAI) for measuring the volume and FAI of PCAT. The target coronary vessel and surrounding adipose tissue were segmented by the software. Lesion FAI (FAI_lesion_) was defined as the area from the proximal to the distal segment of the lesion without plaques. The PACT surrounding the lesion was sampled by radially extending 1 × the vessel wall diameter, and the attenuation was measured in Hounsfield Units (HU) within the range of -190 HU to -30 HU [15]. To minimize the influence of non-adipose tissue, small surrounding branches and coronary veins were manually excluded (Fig. 2). Two experienced diagnostic physicians, blinded to other examination results, independently measured the above parameters, and the average lesion FAI values between the two observers were used for further analysis.

#### CT-FFR

**Fig. 3:**
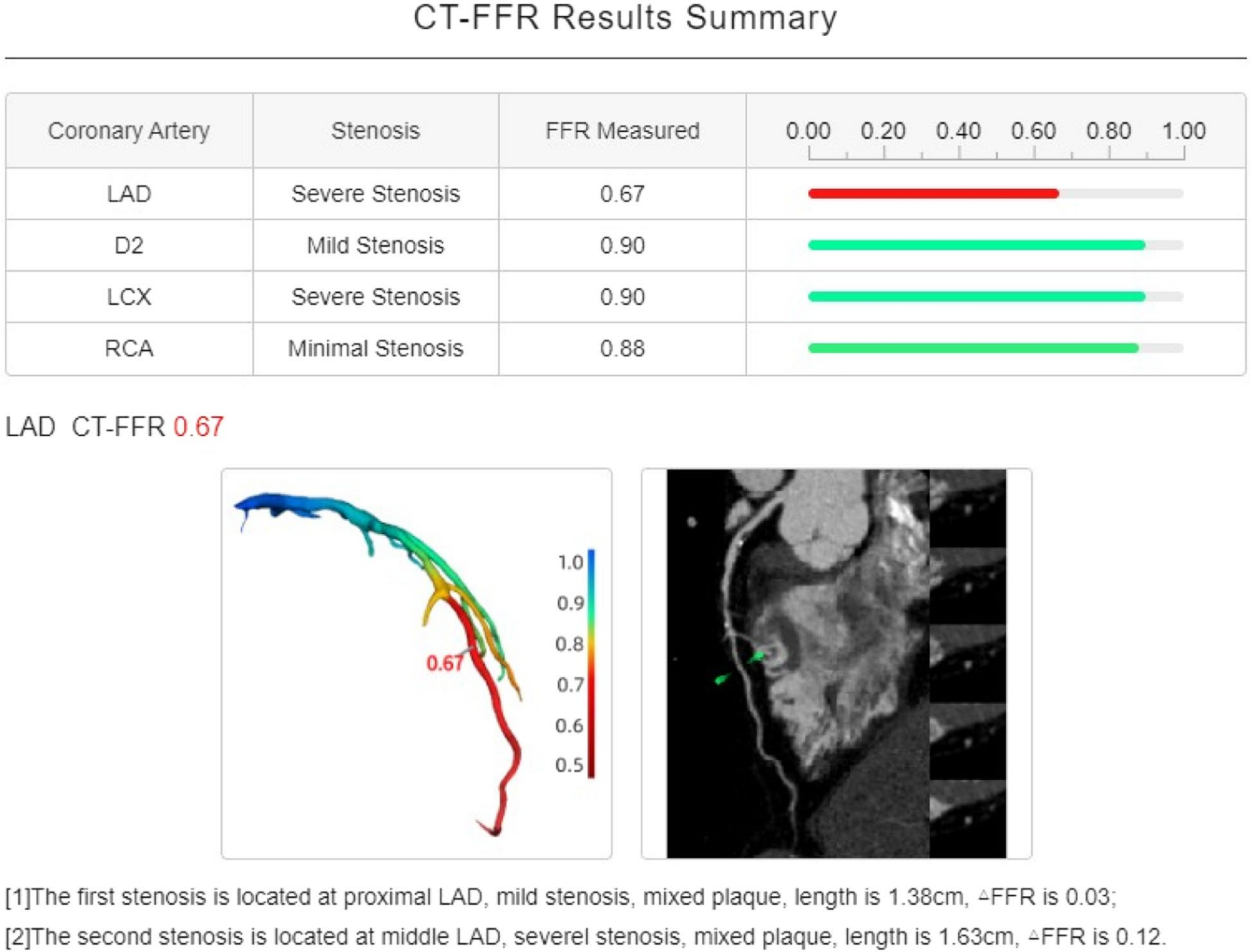
Measurement of Lesion CT-FFR. The measurement table indicated that the CT-FFR value of the LAD blood vessel was 0.67, the lower left 3D reconstructed color map indicated that the LAD was the responsible blood vessel for ischemia, and the lower right blood vessel analysis map indicated that there were mixed plaques in the near and middle part of the LAD, and the responsible plaque was located in the middle part with a length of 1.63 cm

To assess the minimum CT-FFR and ΔCT-FFR, the SSF2-reconstructed CCTA images were used with an AI-assisted coronary diagnostic system and AI-based fractional flow reserve measurement system. The minimum CT-FFR refers to the CT-FFR value measured within a 20 mm range distal to the most severe stenosis, and ΔCT-FFR refers to the difference between the CT-FFR values measured within 20 mm proximal and distal to the most severe stenosis [16]. A CT-FFR value of ≤ 0.8 was considered indicative of hemodynamic abnormalities (Fig. 3). Two experienced diagnostic physicians, blinded to other results, independently performed CT-FFR simulations, and the average value of the measurements was used for further analysis.

### Statistical analysis

SPSS 26.0 was used to perform statistical analyses of the collected clinical and imaging data. Variables with normal distribution were presented as mean ± standard deviation (x ± SD). Comparisons between two groups were examined using independent t-tests, and one-way ANOVA was used for comparisons across multiple groups. For non-normally distributed variables, data were presented as the median (25th percentile, 75th percentile). The Mann–Whitney U test was used for comparisons between two groups, and the Kruskal–Wallis rank-sum test was applied for comparisons across multiple groups. Correlations between two continuous variables were assessed using Pearson’s correlation coefficient or Spearman’s correlation coefficient. A *P*-value of < 0.05 was considered statistically significant.

## Result

### Baseline

Based on the CTA or coronary angiography results, the included 207 vessels with lesions were classified according to the CAD-RADS classification standard: stenosis < 50% (128 vessels) and stenosis > 50% (79 vessels). Patients with more than one lesion with stenosis > 50% were classified into the myocardial ischemia group (44 cases), and the rest were classified as the non-myocardial ischemia group (86 cases). In patients with multiple lesions, the site with the minimum CT-FFR value was used to represent the patient’s hemodynamic status for statistical analysis. Comparisons of general information between the two groups showed that the average age of patients in the myocardial ischemia group was significantly higher than that in the non-myocardial ischemia group (*P* < 0.001). There were no significant differences between the two groups in sex or cardiovascular risk factors (*P* > 0.05), as shown in Table 1.

**Table 1:** Baseline characteristics between myocardial ischemia group and non-myocardial ischemia group

Clinical Characteristics	Overall (*n* = 130)	Myocardial Ischemia Group (*n* = 44)	Non-Myocardial Ischemia Group (*n* = 86)	*p-value*
Age (years)	63.5 ± 8.75	65.2 ± 4.31	57.3 ± 4.24	< 0.001
Male (n, %)	94(72.3%)	32(72.8%)	62(72.1%)	0.738
Cardiovascular Risk Factors (n, %)				
Hypertension	46(35.4%)	15(34.1%)	31(36.0%)	0.695
Diabetes	42(32.3%)	12(27.3%)	30(34.9%)	0.086
Hyperlipidemia	37(28.5%)	10(22.7%)	27(31.4%)	0.057
Smoking History	32(24.6%)	10(22.7%)	22(25.6%)	0.603

*Note: P* < 0.05 indicates statistically significant differences

### Comparison of coronary artery stenosis distribution, CT-FFR, and FAI

Table 2 shows the comparison of coronary artery stenosis distribution, CT-FFR, and FAI values between vessels with < 50% stenosis and with > 50% stenosis. There were no significant differences in the distribution of stenotic vessels between the two groups. The minimum CT-FFR was higher in vessels with < 50% stenosis than in vessels with > 50% stenosis, while ΔCT-FFR was lower in vessels with < 50% stenosis than in vessels with > 50% stenosis. FAI-LAD, FAI-LCX, FAI-RCA, and FAI-Mean were all higher in vessels with > 50% stenosis compared to vessels with < 50% stenosis (*P* < 0.05). There were no statistically significant differences in PCAT and total PCAT volume in LAD, LCX, and RCA between the two groups.

**Table 2:** Comparison of coronary artery stenosis distribution, CT-FFR, and FAI parameters

Clinical Characteristics	Vessels with stenosis < 50% (*n* = 128)	Vessels with stenosis > 50% (*n* = 79)	*p-value*
Vessel			0.351
LAD	57(44.5%)	30(38.0%)	
LCX	42(32.8%)	28(35.4%)	
RCA	29(22.7%)	21(26.6%)	
Minimum CT-FFR	0.86 ± 0.11	0.69 ± 0.08	< 0.001
ΔCT-FFR	0.09 ± 0.02	0.25 ± 0.06	< 0.001
FAI-LAD(HU)	-89.12 ± 8.85	-85.34 ± 8.52	0.021
FAI-LCX(HU)	-86.38 ± 8.63	-81.56 ± 7.68	0.018
FAI-RCA(HU)	-95.32 ± 9.02	-87.11 ± 8.90	0.013
FAI-Mean(HU)	-88.41 ± 7.89	-83.34 ± 7.76	0.008
LAD-PCAT Volume(mm^3^)	1276.57 ± 342.28	1326.64 ± 398.87	0.695
LCX-PCAT Volume(mm^3^)	961.63 ± 229.02	945.34 ± 227.18	0.773
RCA-PCAT Volume(mm^3^)	1673.52 ± 428.36	1605.22 ± 425.12	0.536
Total PCAT Volume(mm^3^)	4174.68 ± 857.84	4125.45 ± 841.27	0.848

### Correlation between CT-FFR, FAI, and the degree of coronary artery stenosis

Table 3 shows the correlation between CT-FFR, FAI values, and the degree of coronary artery stenosis. A negative correlation was observed between the minimum CT-FFR in the three main coronary artery branches and the CAD-RADS classification, while both ΔCT-FFR and FAI values were positively correlated with the CAD-RADS classification (*P* < 0.05).

**Table 3:** Correlation between CT-FFR and FAI parameters of coronary artery branches and the degree of coronary stenosis

	CAD-RADS Classification
LAD	
minimum CT-FFR	-0.483
ΔCT-FFR	0.502
FAI(HU)	0.339
LCX	
minimum CT-FFR	-0.647
ΔCT-FFR	0.704
FAI(HU)	0.591
RCA	
minimum CT-FFR	-0.628
ΔCT-FFR	0.585
FAI(HU)	0.463

### Plaque characteristics between vessels with > 50% stenosis and vessels with < 50% stenosis

The median CT-FFR value was significantly lower in vessels with > 50% stenosis compared to vessels with < 50% stenosis (*P* < 0.001). Non-calcified plaques were more prevalent in vessels with > 50% stenosis, located mostly in the LAD, whereas calcified plaques were more common in vessels with < 50% stenosis (*P* < 0.001). In addition, plaques in vessels with > 50% stenosis were longer, with a greater degree of luminal stenosis, and both total plaque volume and total plaque burden were larger than those in vessels with < 50% stenosis (*P* < 0.001). The FAI_lesion_ value was higher in vessels with > 50% stenosis compared to vessels with < 50% stenosis (*P* < 0.001) (Table 4).

**Table 4:** Comparison of plaque characteristics and PCAT parameters between vessels with stenosis > 50% and vessels with stenosis < 50%

Plaque Characteristics	Vessels with Stenosis > 50%(*n* = 79)	Vessels with Stenosis < 50%(*n* = 128)	*p-value*
Median CT-FFR	0.68(0.53, 0.77)	0.84(0.65, 0.92)	< 0.001
Plaque Types (n, %)			< 0.001
Calcified Plaques	26(32.9)	61(47.7)	
Mixed Plaques	23(29.1)	37(28.9)	
Non-Calcified Plaques	30(38.0)	30(23.4)	
Plaque Distribution (n, %)			< 0.001
LAD	43(54.4)	39(30.5)	
LCX	16(20.3)	45(35.2)	
RCA	20(25.3)	44(34.4)	
Plaque Length (mm)	19.35(8.90, 25.35)	13.20(8.10, 20.95)	< 0.001
Minimum Lumen Area (mm^2^)	2.40(1.40, 4.30)	5.15(3.75, 5.10)	< 0.001
Maximum Stenosis Degree (%)	67(53, 78)	48(35, 60)	< 0.001
Total Plaque Volume (mm^3^)	275.00(160.85, 340.00)	163.50(100.30, 225.50))	< 0.001
Lipid Component Volume Percentage (%)	26.37(20.32, 30.72)	28.25(21.54, 34.21)	0.073
Fibrous Component Volume Percentage (%))	40.26(33.42, 49.73)	34.21(27.44, 43.56)	0.062
Calcified Component Volume Percentage (%)	37.24(30.17, 47.48)	40.26(32.33, 48.84)	0.109
Plaque Burden (%)	63.30(59.40, 71.45)	59.10(55.95, 68.20)	< 0.001
Calcified Burden (%)	22.14(12.15, 29.10)	21.60(11.40, 27.80)	0.527
FAI_lesion_(HU)	-76.26 ± 8.18	-88.54 ± 8.73	< 0.001
Pericoronary Adipose Tissue (PCAT) volume (mm^3^)	781(429, 1599)	806(432, 1648)	0.936

## Discussion

The main finding of this study is that the FAI value in flow-limiting lesions is significantly higher than in non-flow-limiting lesions. CT-FFR measurement system can meet this clinical demand. This system is a new AI technology based on coronary CTA image data. The application of this technology can realize the comprehensive information of coronary heart disease patients' structure and function in only one CTA examination, which makes up for the limitations of multiple examinations in the past. And the technology has the advantages of convenient operation, non-invasive, and does not increase the extra cost of patients and load drugs, which is conducive to clinical application. With our initial hypothesis that FAI may be a new parameter indicating hemodynamic changes in coronary artery stenosis, FAI and CT-FFR provided additional value in identifying hemodynamically significant lesions. This may be due to several factors. FAI is considered an important indicator of edema in perivascular adipose tissue, likely caused by the release of various cytokines from active vascular inflammation of coronary plaques [17]. Paracrine inflammatory signals from the vessel wall may influence biological processes such as adipocyte differentiation, proliferation, and lipolysis, which can inhibit lipid accumulation [18]. Therefore, in cases of acute vascular inflammation, the density of perivascular adipose tissue increases due to inhibited lipid accumulation. From previous studies, vascular inflammation is considered a factor contributing to endothelial dysfunction [19]. If coronary lesions are associated with impaired vasodilation in the stenotic site and the degree of dilation is similar to other parts of the vessel, a relative pressure drop occurs during adenosine-induced maximal hyperemia, resulting in a positive FFR value. Therefore, vessels with more severe stenosis have a higher FAI value, representing a greater degree of vascular inflammation. Accordingly, the diagnostic value of FAI combined with CT-FFR for hemodynamically significant lesions is comparable to the accuracy of CCTA, making FAI and CT-FFR effective combined parameters for excluding ischemic coronary stenosis. The sensitivity of FAI in identifying flow-limiting lesions is high, but its specificity is relatively lower [20]. When combining FAI with CT-FFR, the diagnostic performance for predicting hemodynamically significant lesions can be significantly improved, increasing specificity while maintaining high sensitivity [21, 22].

Moreover, the correlation between FAI and plaque composition found in this study suggests that different plaque components can lead to varying degrees of inflammation in the surrounding adipose tissue. A previous study suggests that a higher perivascular FAI value is associated with higher levels of inflammatory expression [23]. During the early stages of atherosclerosis, the deposition of low-density lipoproteins and aggregation of inflammatory cells induce the formation of a focal inflammatory microenvironment, which inhibits preadipocyte differentiation and lipid accumulation in surrounding adipose tissue, resulting in smaller adipocytes, reduced fat content, and increased water content [24]. As the necrotic core burden in non-calcified plaques gets larger, plaques get less stable, and surrounding inflammation gets greater. Therefore, higher FAI values can be observed around non-calcified plaques with higher levels of inflammation [25].

In this study, CT-FFR with the narrowest coronary branch was found to be negatively correlated with CAD-RADS classification. ΔCT-FFR and FAI were positively correlated with CAD-RADS classification (*P* < 0.05), which was similar to previous studies. The basic pathological cause of acute coronary syndrome is the formation of mixed plaques or soft plaques in the intima of arteriosclerosis vessels, resulting in severe stenosis or complete occlusion of the diseased vessels [26]. CT-FFR is based on CCTA images and then reconstructed into three-dimensional coronary artery images, supplemented by special computer software to provide simulated FFR results. CT-FFR in the application of AI technology is a non-invasive method based on high-quality coronary CTA image data without loading drugs, which is a new method for noninvasive evaluation of coronary FFR. Studies have shown that the FFR value measured by CT-FFR has a good consistency with the traditional invasive FFR value [27]. CT-FFR has a high diagnostic efficacy for ischemic coronary artery stenosis, a certain prognostic value for high-risk plaques, and a predictive value for coronary plaque characteristics and subsequent cardiac events [28]. Studies have shown that CT-FFR correctly reclassified 68% of false-positive CCTA patients and 67% of false-positive CCTA vessels as true negative results, reducing the clinical misdiagnosis rate [29].

Traditional high-risk plaque features, such as LAP, NRS, PR, and spotty calcifications, are unrelated to hemodynamic status [30, 31]. However, there has been controversy regarding the significance of plaque features and hemodynamic indicators in coronary artery lesions, as other CCTA studies have shown that the presence of plaque necrosis and total LAP volume may lead to impaired myocardial perfusion [32]. Therefore, further research is needed to explore the relationship between plaque characteristics and coronary blood flow function.

In this study, after grouping according to the degree of arterial stenosis, it was found that the incidence of calcified plaque, vulnerable plaque and multi-vessel coronary artery disease in the stenosis > 50% group was higher than that in the stenosis < 50% group, and the CT-FFR value was significantly lower than that in the stenosis < 50% group. The coronary structural characteristics of patients with severe arterial stenosis are mainly characterized by calcified plaques, vulnerable plaques and multi-vessel lesions, which is consistent with the results of previous studies [27]. Patients with severe arterial stenosis have more significant hemodynamic changes, and their proportion of acute coronary syndrome may be higher. The incidence of calcified plaque, vulnerable plaque and myocardial ischemia in patients is positively correlated with the degree of coronary stenosis, which is consistent with previous reports [28].

The present study has some limitations. First, it is a post-hoc analysis of pre-collected data. Enrolled patients presented with symptoms such as exertional chest pain or angina, with an intermediate risk of obstructive CAD. Additionally, due to the exclusion of patients with a low or high risk of obstructive CAD, the existence of selection bias is possible. Therefore, the findings of the present study shall not be applied to such patients, and future studies are needed to compare at an individual level to determine the best method among available approaches.

In conclusion, this study demonstrates that FAI is associated with coronary stenosis and myocardial ischemia, and it may serve as a novel indicator for identifying myocardial ischemia. Additionally, both FAI and CT-FFR show good predictive abilities for severe coronary stenosis.

## Data Availability

The data used to support the findings of this study are available from the corresponding author upon request.
